# Delayed but long-term resolution of nevus of Ota after cessation of picosecond 755-nm alexandrite laser treatments

**DOI:** 10.1016/j.jdcr.2025.06.058

**Published:** 2025-08-06

**Authors:** Angela Moore, An Nguyen, Luke Moore, Ly Nguyen, Stephen Moore, Michael Thornton

**Affiliations:** a Arlington Center for Dermatology, Arlington, Texas; b Arlington Research Center, Arlington, Texas; c Department of Dermatology, Baylor University Medical Center, Dallas, Texas; d Texas Christian University Anne Burnett Marion School of Medicine, Fort Worth, Texas; e McGovern Medical School at UTHealth Houston, Houston, Texas; f Mansfield Cosmetic Surgery Center, Mansfield, Texas

**Keywords:** alexandrite, hyperpigmentation, nevus of Ota, picosecond

## Introduction

Nevus of Ota (ocular dermal melanosis) is a unilateral benign melanosis in V1 or V2 trigeminal distribution.^1^^,^^2^ It appears as a bluish/brownish hyperpigmentation of the eye and adnexa, and it is associated with an increased risk of uveal melanoma^1^ and glaucoma.^2^ Q-switched nanosecond lasers were initially reported for the treatment of nevus of Ota and other dermal pigmented lesions.^3^ More recently, picosecond lasers with 755-nm alexandrite, 694-nm ruby, and 1064-nm neodymium-doped yttrium aluminum garnet have been shown to be more effective than Q-switched nanosecond lasers for nevus of Ota treatment.^4^, ^5^, ^6^, ^7^ In these cases, treatment until clinical clearance was performed.

## Case report

**Fig 1:**
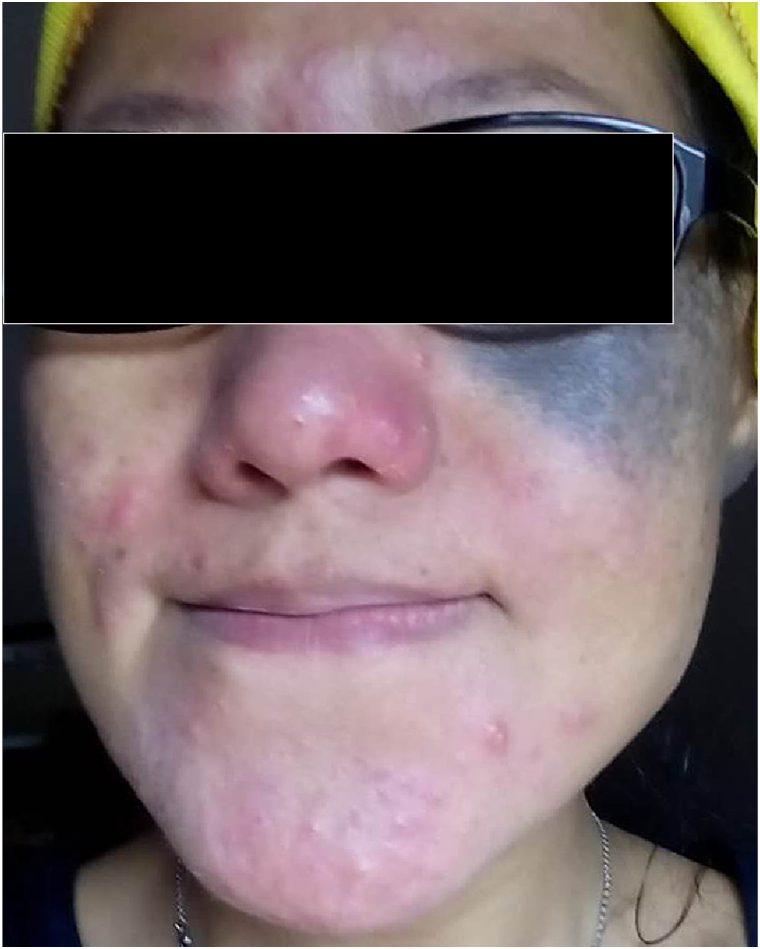
Left medial malar cheek and left ear with nevus of Ota prior to treatment.

A 35-year-old Asian woman with a bluish-gray confluent pigmentation along the left V2 distribution along the medial malar cheek, zygomatic arch, and left ear (Fig 1) was treated with a 750-picosecond 755-nm alexandrite laser with a 3.2 to 3.5 mm spot size, 2.08 to 2.49 J/cm^2^ fluence, 10 Hz, and 1179 to 2210 pulses every 2 weeks for 6 sessions. Preoperative topical 20% benzocaine/6% lidocaine/4% tetracaine mixture was applied for 30 minutes prior to each session. Postoperative physical sunblock with 11% zinc oxide was applied immediately after the procedure and daily every morning.

**Fig 2:**
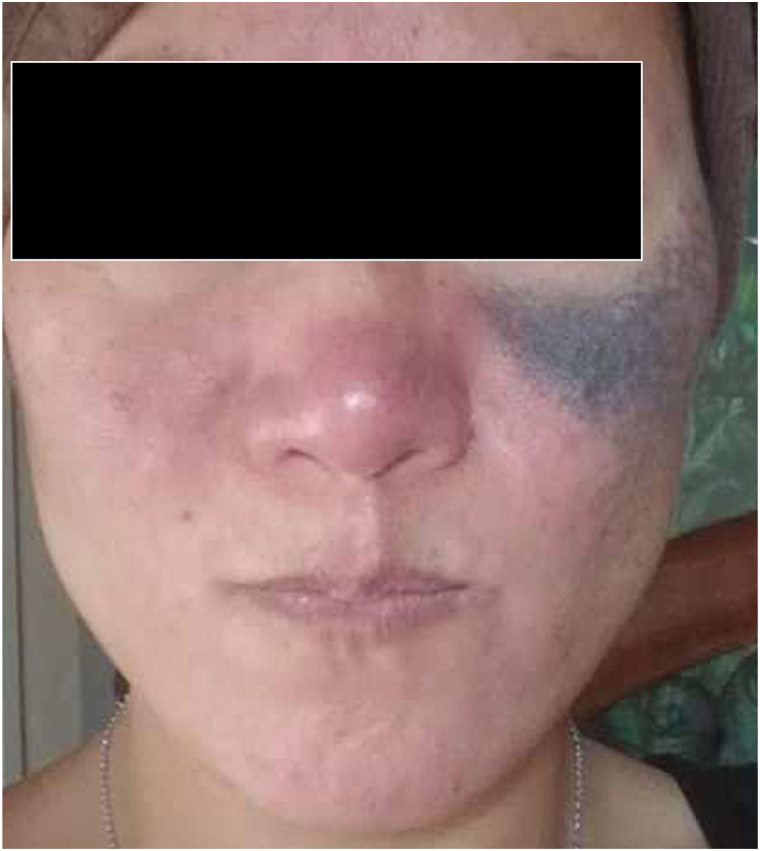
Nevus of Ota after 6 picosecond alexandrite laser sessions.

Treatment terminated when the patient moved after 12 weeks despite persistent central blue-black pigmentation after session 6 (Fig 2). Local skin reactions included pain of 3 to 4 of 10 during treatment, immediate physician-observed mild erythema during treatment, and transient self-reported erythema for 1 to 2 days after each session. No hyperpigmentation or hypopigmentation was noted.

**Fig 3:**
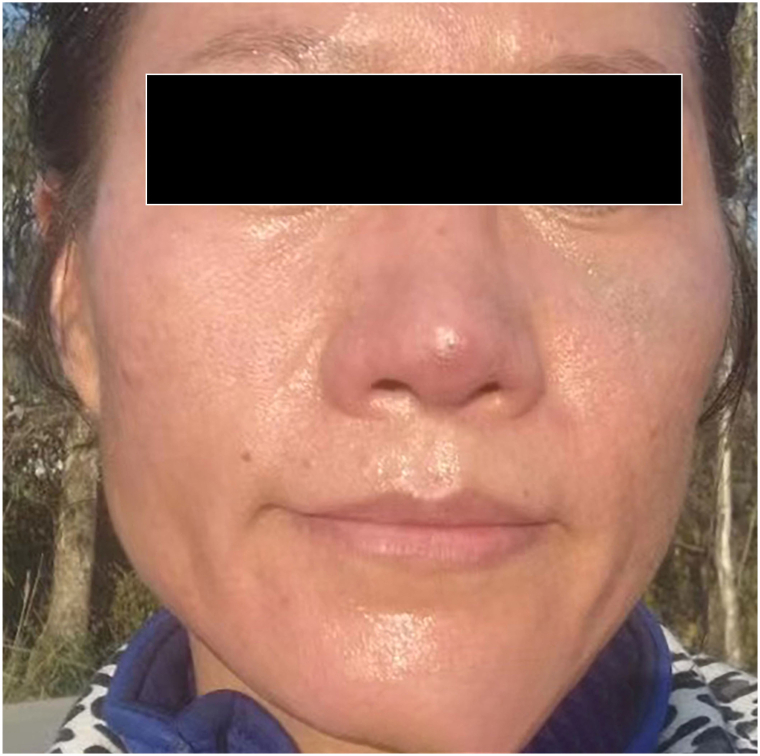
Left medial malar cheek and ear 6 months after completion of alexandrite picosecond laser sessions.

Progressive clinical improvement and resolution of the nevus of Ota occurred over the 6 months following the final treatment session (Fig 3). The nevus of Ota did not recur for 5 years after the laser treatments, as documented by follow-up email photos from the patient.

## Discussion

Histologically, the blue-gray pigmentation in nevus of Ota results from dermal dendritic melanocytes with melanin granules and melanophages scattered in the upper and lower dermis.^1^^,^^2^ Picosecond lasers rely on photomechanical effects rather than photothermal effects by generating photoacoustic destruction of the melanin granules and melanophages with a decreased photothermal component.^4^^,^^8^ Clinical resolution of hyperpigmentation hypothetically occurs as macrophages carry away the fractured particles even after cessation of picosecond laser sessions.^4^^,^^8^ Only 1 other article in the literature reported delayed clearance of nevus of Ota after cessation of treatment with a 1064-nm Q-switched neodymium-doped yttrium aluminum garnet laser.^9^

In our patient, the continued clinical improvement of the nevus of Ota occurring over the next 6 months following cessation of picosecond alexandrite laser sessions further supports the postulated theory of macrophagic activity in eliminating fractured pigment particles. Further study is warranted in treating nevus of Ota with less frequent sessions, leading to fewer picosecond laser treatment sessions in total.

## Conflicts of interest

None disclosed.
